# Effects of preoperative oral carbohydrates on patients undergoing ESD surgery under general anesthesia: A randomized control study

**DOI:** 10.1097/MD.0000000000015669

**Published:** 2019-05-17

**Authors:** Yan Wang, Zhenqiang Zhu, Hui Li, Yaqi Sun, Guohao Xie, Baoli Cheng, Feng Ji, Xiangming Fang

**Affiliations:** a Department of Anesthesiology; b Department of Digestive Medicine, The First Affiliated Hospital, College of Medicine, Zhejiang University, Hangzhou, China.

**Keywords:** carbohydrates, ERAS, ESD, gastric peristalsis, VAS

## Abstract

**Background:**

Preoperative oral carbohydrate (POC) has been recommended as an important element of the enhanced recovery after surgery (ERAS) protocol, but its effect on patients undergoing endoscopic submucosal dissection (ESD) remains unclear. Our study aims to investigate the effects of POC for ESD surgery, with particular focus on perioperative well-being and gastric peristalsis.

**Methods:**

A prospective, randomized, and controlled study of patients undergoing ESD was conducted. Seventy-three patients were assigned to 2 groups: experiment (36 patients) and control (37 patients). The experiment group received oral carbohydrate solution 710 mL the night before and 355 mL 2 hours prior to operation. The control group fasted for 10 hours prior to operation. Gastric empty assessment, peristaltic score, and operation score were measured. In addition, visual analogue scale (VAS) scores for 6 parameters (thirst, hunger, mouth dryness, nausea, vomit, and weakness) of wellbeing were compared perioperatively. Preoperative basic conditions of patients, postoperative complications, and their clinical outcomes were also recorded.

**Results:**

Before anesthesia induction, gastric sonography score was higher in experiment group, while sucked fluid by gastroscopy was similar between 2 groups. And no patient had regurgitation. Moreover, gastric peristaltic score and operation score before operation were both lower in experiment group. Importantly, VAS scores for 3 parameters (thirst, hunger, and mouth dryness) were significantly lower in experiment patients. In addition, clinical outcomes including first time exhaust, first time for drinking water, the usage of hemostasis, postoperative complication, lengths of hospital stay, and in-hospital expense were not significantly different between 2 groups.

**Conclusions:**

Oral administration of carbohydrates preoperatively instead of fasting improves the feelings of thirst, hunger, and mouth dryness in patients following ESD surgery without enhancing risk of regurgitation. And, avoiding preoperative fasting with POC can decrease the degree of gastric peristalsis that may facilitate the successful completion of ESD surgery.

## 1 Introduction

Endoscopic submucosal dissection (ESD) has been regarded as an effective and minimally invasive endoscopic treatment for early gastric cancer. The Enhanced Recovery After Surgery (ERAS), which consists of minimally invasive surgery, preoperative nutrition, avoidance of perioperative fasting, and early mobilization, has been shown to attenuate surgical trauma and reduce postoperative complication in various surgeries.^[1,2]^ Intake of solid food up to 6 hours and clear fluids up to 2 hours before induction of anesthesia has been suggested in the ERAS protocol. Preoperative oral carbohydrates (POC), one of the main elements of the ERAS protocol, has beneficial effect on minimizing preoperative fasting and improving patients’ feelings of thirst, hunger, and anxiety in the perioperative period.^[3–7]^ POC, with high energy content, does not pose any threat from vomiting or aspiration when taken 2 hours before anesthesia.^[8–10]^ Studies have also revealed that avoiding preoperative fasting by providing a carbohydrate drink before surgery can attenuate the magnitude of postoperative insulin resistance, reduce the nitrogen losses, and improve muscular strength, which result in better clinical outcomes.^[7,11]^ Several studies have reported the impact of POC in patients undergoing open abdominal surgery, thoracic surgery, and orthopedic surgery. However, as far as we know, no study investigates the effect of POC in patients with ESD surgery. We therefore conducted a randomized control study to assess the effect of POC in contrast to fasting on gastric emptying, gastric peristalsis, perioperative well-being, and clinical outcomes, in patients undergoing ESD surgery, further getting an insight into the application of ERAS in ESD.

## 2 Methods

### 2.1 Patients

This investigation was designed as prospective, randomized controlled study. A total of 116 patients, aged 18 to 80 years, undergoing general anesthesia for elective ESD operation in The First Affiliated Hospital, College of Medicine, Zhejiang University from July 2017 to December 2017 were assessed for eligibility. Indication for ESD was early gastric cancer, gastric stromal tumor, gastric mucosal lesion, and esophagus mucosal lesion. Inclusion criteria include patients with the American Society of Anesthesiologists (ASA) class I to II; BMI 18.5–23.9; no serious heart disease; no lung disease. Exclusion criteria include patients with abnormal or impaired gastrointestinal motility; gastric emptying delay disease, such as pyloric obstruction; high risk of regurgitation and aspiration, such as gastroesophageal reflux; severe malnutrition or severe anemia (serum albumin less than 35 g/L and hemoglobin less than 90 g /L); hydroelectrolyte disturbance; diabetes or abnormal glucose tolerance; abnormal endocrine hormone or taking steroid medication recently; moderate or severe lung function impairment; pregnancy or lactating women during perioperative period. One hundred patients were selected for randomization.

### 2.2 Randomization and masking

Enrolled patients were randomized into 1 of 2 groups: experiment group (received oral carbohydrate solution (3% energy, 5% carbohydrate, and 2% sodium) 710 mL the night before and 355 mL 2 hours prior to gastroscopy examination before anesthesia induction, and control group (fasted for 10 h prior to gastroscopy examination). We used random number table method to divide enrolled patients into control group (n = 50) and experiment group (n = 50). On operation day, anesthesiologists were blinded after assignment to interventions. Twenty-seven patients were excluded due to long operation time (>4 h), cancelled operation, unsuccessful operation, sent to intensive care unit or transferred to open operation during surgery. Seventy-three patients were eligible for inclusion among whom 36 patients belong to experiment group and 37 patients belong to control group. This study obtained the written consent from all patients before entering the study. The Ethics committee of The First Affiliated Hospital, College of Medicine, Zhejiang University approved the trial.

### 2.3 Anesthesia methods

In the operating room, patients were regularly monitored heart rate, blood pressure, electrocardiogram, and SpO_2_. All patients were given standard mask with 3L/min oxygen in operation room. Anesthesia was induced in patients with intravenously giving midazolam 0.04 mg/kg, sufentanil 0.4 ug/kg, vecuronium bromide 0.1 mg/kg, and propofol 1.5 to 2 mg/kg. Then, endotracheal intubation was performed and the tube was linked to the ventilator to maintain positive pressure ventilation with pure oxygen flow 1.5 L/min, tidal volume 8 mL/kg, breathing frequency 12 times/min, and inspiration and expiration ration 1:2 in order to keep SpO_2_ > 95% and PetCO_2_ between 30 and 45 mm Hg. Propofol at the rate of 3 to 9 mg kg^−1^·h^−1^, cisatracurium 2 to 3 ug kg^−1^·min^−1^, and remifentanil 0.2 ug kg^−1^·min^−1^ were continued to maintain anesthesia. And sufentanil was intermittently administered during surgery. Heart rate, electrocardiogram, SpO_2_, PetCO_2_, and bispectral index (BIS) between 40 and 60 were recorded during operation. 0.2 to 0.5 mg of atropine was given once heart rate was lower than 50 per min. 6 mg ephedrine was injected when blood pressure was lower than 90/60 mm Hg or decreased 30% of the basic value. If blood pressure was higher than 160/100 mm Hg and the effects of anesthesia depth and surgical complications were excluded first, urapidil was injected. Anesthesiologist observed the changes of airway pressure and PetCO_2_ and paid attention to the occurrence of intraoperative complications including perforation, subcutaneous, and mediastinal emphysema.

### 2.4 Monitoring index

The primary outcome measure was VAS scores for 6 parameters (thirst, hunger, mouth dryness, nausea, vomit, and weakness) of wellbeing. Secondary outcomes were gastric empty assessment, peristaltic score, operation score, and patients’ outcomes.

### 2.5 Gastric empty assessment before anesthesia induction

Gastric sonography is a reliable diagnostic tool to assess gastric content and volume.^[12–16]^ A standardized gastric scanning protocol was applied before anesthesia induction. We proposed a 3-point grading system based on qualitative sonographic assessment of the antrum in the supine and right lateral positions that correlates well with predicted gastric volume. Patients were classified as followings: Grade 0 means empty antrum on both supine and right lateral positions, corresponding to a completely empty stomach; Grade 1 means minimal fluid volume detected only in the right lateral position, suggesting a negligible fluid volumes mostly less than 100 mL; Grade 2 means antrum clearly distended with fluid visible in both supine and right lateral positions, correlating with significantly higher fluid volumes (>100 mL) and higher risk of regurgitation of gastric contents on anesthesia induction.^[17]^ In addition, all patients were performed gastroscopy to collect and clear residual fluid in the stomach before anesthesia induction. Lidocaine gel and midazolam 0.03 mg/kg were given prior to gastroscopy to ensure the adaption of patient to the procedure and to decrease anxiety and discomfort during the procedure. During gastroscopy examination, the patients were placed in the left lateral position and the residual fluid was pumped and collected through the collection bottle. After sampling, the stomach was emptied by aspiration and the volume measured.

### 2.6 Gastric peristaltic score and operation score before operation

Gastric peristalsis was evaluated using a 4-grade scale: Grade 1-No peristalsis; Grade 2-Mild peristalsis that peristaltic wave is formed without reaching the pyloric ring; Grade 3-Moderate peristalsis that a pronounced peristaltic wave is formed and reaches the pyloric ring; Grade 4-Vigorous peristalsis that peristaltic wave is deep and strangulating the antrum.^[18]^ We designed an operation 4-grade scale accordingly: Grade 1-No peristalsis that does not affect operation; Grade 2-Mild peristalsis that does not affect operation; Grade 3-Moderate peristalsis that affects operation but operation could continue; Grade 4-Vigorous peristalsis that operation could not continue. Operation score was evaluated by ESD operator before surgery. When operation score is more than 2, intravenous injection of antispasmodic agents is required.

### 2.7 VAS score

In this study, primary outcomes included patient's subjective feeling of well-being. The feelings of thirst, hunger, mouth dryness, nausea, vomit, and weakness were assessed using VAS scores 0 to 10 before anesthesia induction, after extubation, and after sending back to ward.^[19]^ Score 0 means patients have no discomfort at all and score 10 presents patients have the most severe discomfort. Higher score means more severe discomfort. All VAS scores were guided by a blinded investigator.

### 2.8 Clinical outcomes and postoperative complication

In addition, we documented the convalescence condition including first time exhaust, first time for drinking water, the usage of hemostasis, the length of hospital stay (LOS), hospitalization expense, and postoperative bleeding rate and fever rate (temperature > 37.5^o^C).

### 2.9 Statistical analysis

Based on our previous experience, 59% patients with normal fasting before operation have no or mild peristalsis and 93% patients with POC have no or mild peristalsis, a sample size of 54 participants (27 per group) would be required to have 90% power, assuming a Type 1 error of 5%. Assuming 10% of participants would drop out, a minimum sample size of 60 participants was established. SPSS 19.0 statistical software was used to analyze data. Continuous variables were presented as mean ± standard deviation (SD) and categorical variables were shown as number (n) and percentage (%). Independent sample *t* test and χ^2^ test were used to compare any significant change between 2 groups. *P* < .05 was regarded as statistically significant.

## 3 Results

### 3.1 Basic characteristics of patients

As shown in Table 1, age, gender, body weight, ASA classification, intraoperative fluid infusion, intraoperative perforation rate, duration of surgery, and the number of patients receiving atropine or ephedrine had no statistical significance between 2 groups (*P* > .05). In addition, the dose of atropine, ephedrine, sufentanil, and remifentanil given to patients during surgery was not significantly different between 2 groups.

**Table 1:**
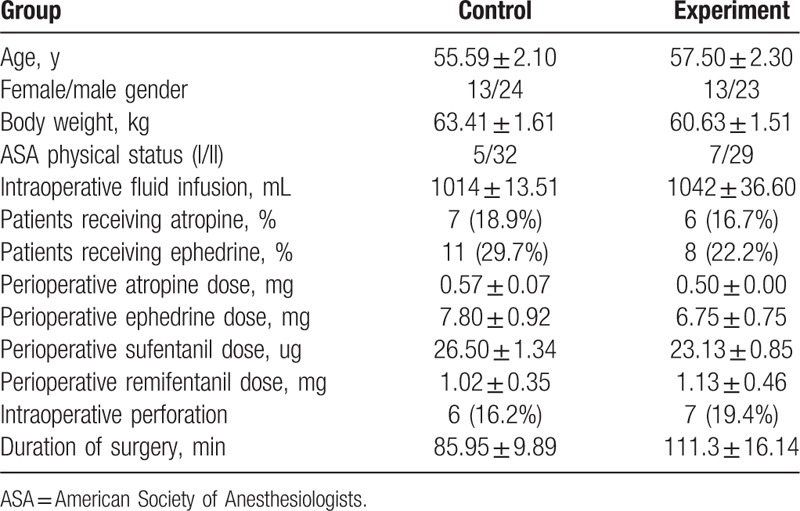
Basic characteristics of patients.

### 3.2 Evaluation of residual fluid in the stomach before anesthesia induction

The evaluation of residual fluid in the stomach includes the gastric sonography grade assessment and sucked fluid measurement by gastroscope before anesthesia induction. Compared with control group, patients in experiment group had higher gastric sonography score (Table 2). No patient reached a residual volume of 100 cc in both groups. No difference in the volume of sucked fluid by gastroscope was found between the groups (Table 2). Importantly, no patient in either control group or experiment group had an episode of regurgitation of gastric content at the time of anesthesia induction.

**Table 2:**
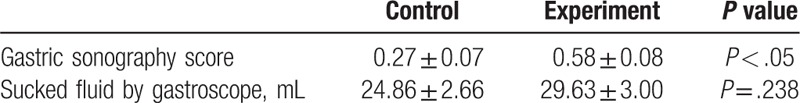
Evaluation of residual fluid in the stomach before anesthesia induction.

### 3.3 Gastric peristaltic score and operation score assessment before operation

**Figure 1:**
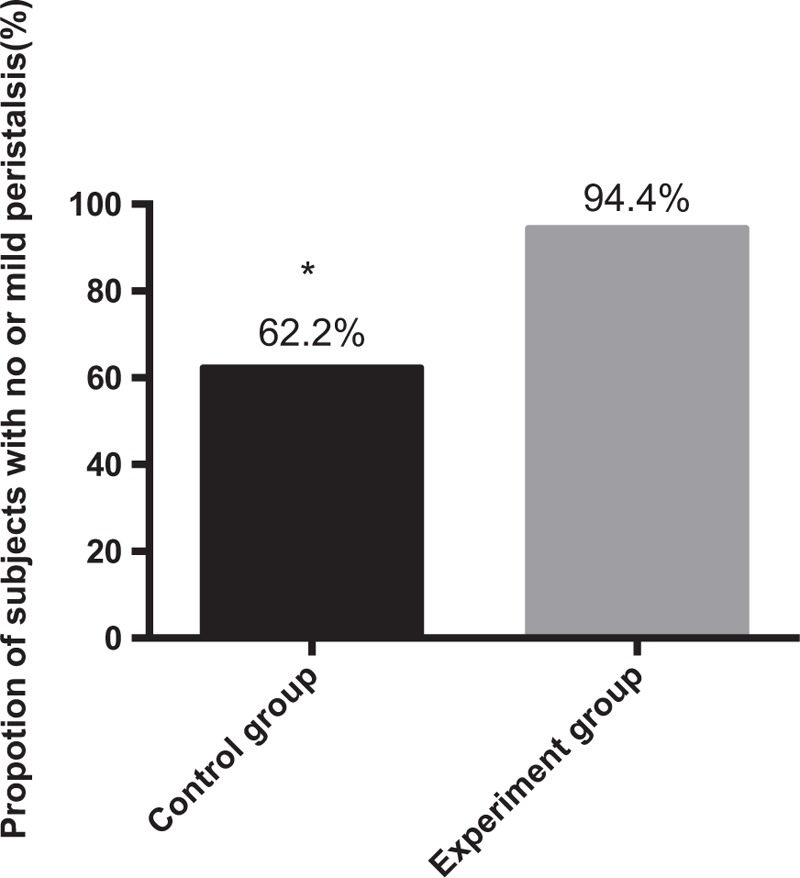
Proportions of subjects with no or mild peristalsis (grade 1 or 2) in 2 groups. ∗*P* < .05.

As shown in Figure 1, in the control group, the proportion of subjects with no or mild peristalsis (grade 1 or 2) was 62.2% (23/37 subjects) and the proportion with moderate peristalsis (grade 3) was 37.8% (14/37 subjects). In the experiment group, the corresponding value with no or mild peristalsis was 94.4% (34/36 subjects) and 5.6% (2/36 subjects) patients had moderate peristalsis. It can be seen that compared with the control group, the experiment group had higher proportions of subjects with no or mild peristalsis (grade 1 or 2). To see the difference more clearly, we calculated the peristaltic score and found that the peristaltic score of subjects in experiment group was significantly lower than that in control group (Table 3).

**Table 3:**
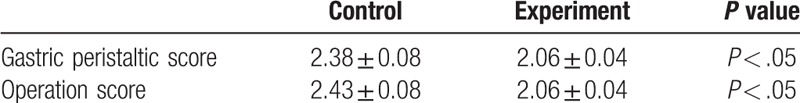
Evaluation of gastric peristaltic score and operation score.

**Figure 2:**
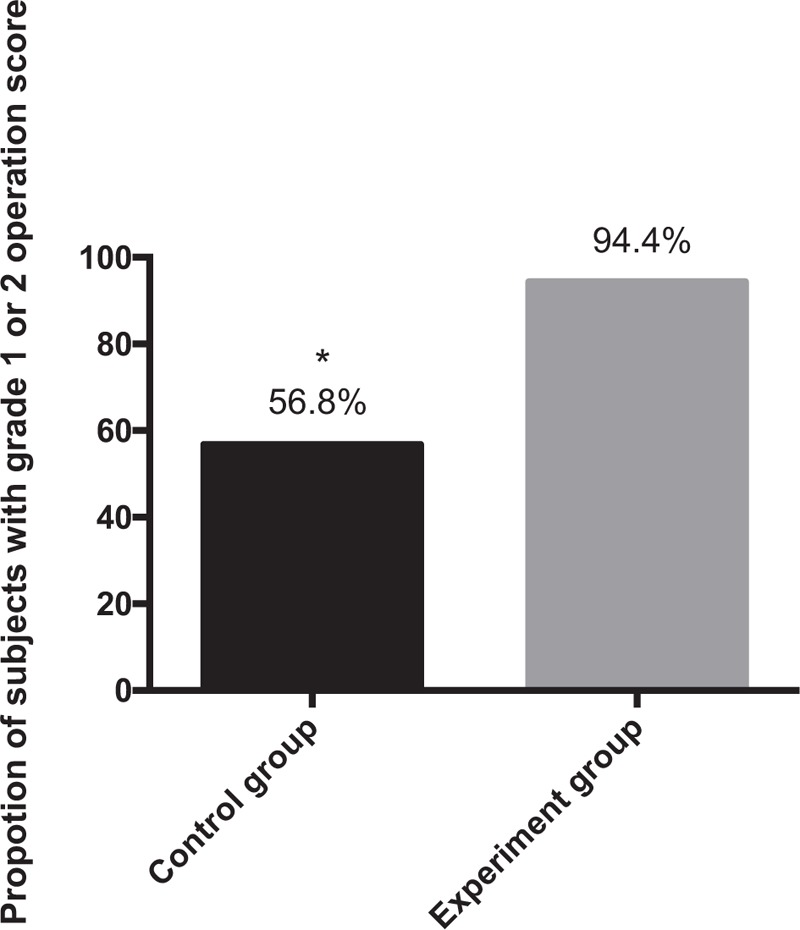
Proportions of subjects with grade 1 or 2 operation score in 2 groups. ∗*P* < .05.

Next, we compared the operation score as shown in Figure 2. In the control group, the proportion of subjects with grade 1 or 2 was 56.8% (21/37 subjects) and the proportion with grade 3 was 43.2% (16/37 subjects). In the experiment group, the corresponding value with grade 1 or 2 was 94.4% (34/36 subjects) and only 5.6% (2/36 subjects) patients had grade 3 score. There was significance in the difference between each group. In addition, the operation score of subjects in experiment group was significantly lower than that in control group (Table 3).

Moreover, once the operation score was grade 3 or grade 4, the need for spasmolytics was given. Therefore, in control group, nearly half (16/37) patients were used with anisodamine during surgery once while in experiment group only 2 patients were injected with anisodamine.

### 3.4 VAS scores assessed before anesthesia induction, after extubation, and after back to ward

In fact, the provision of POC can help alleviate some of the psychological stress associated with ESD surgery. Subjective feelings of discomfort were measured during the perioperative period for 6 parameters using VAS scores (thirst, hunger, mouth dryness, nausea, vomit, and fatigue). We found that before anesthesia induction, the experiment group experienced significantly less thirst, hunger, and mouth dryness compared with control group, whereas no changes were found in nausea, vomit, and fatigue (Table 4). After endotracheal extubation, VAS scores for thirst, hunger, and mouth dryness were significantly lower in experiment group compared to control group. However, no significant difference of VAS scores for nausea, vomit, and fatigue was found among groups. After back to ward, comparing with control group, patients in experiment group had lower VAS scores for thirst, hunger, and mouth dryness, while the VAS sores for nausea, vomit, and fatigue were not significantly different.

**Table 4:**
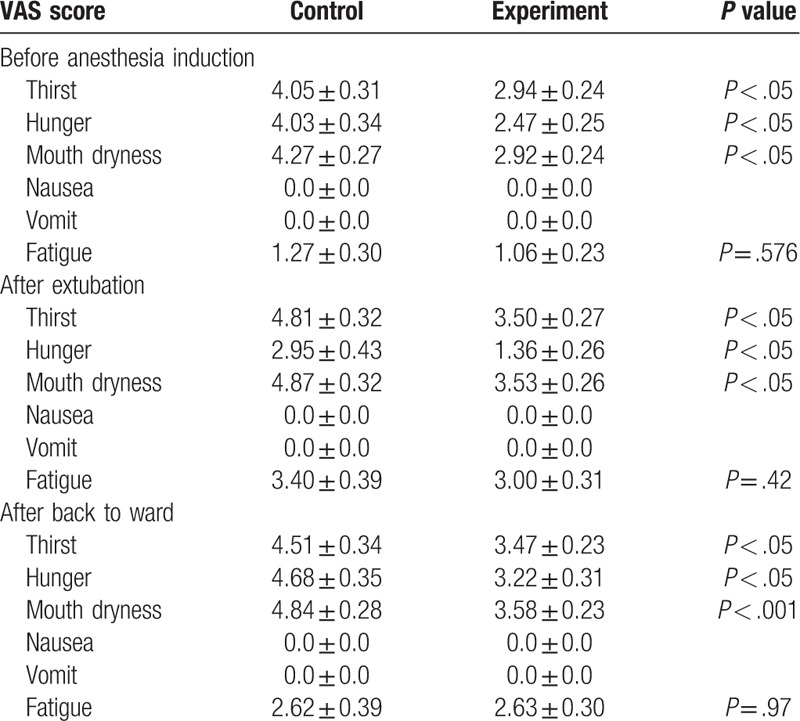
Well-being by VAS score before anesthesia induction, after extubation, and after back to ward.

### 3.5 Convalescence

Summary of postoperative rehabilitation is shown in Table 5. We found no statistically significant differences in the time of first exhaust and drinking water after surgery between 2 groups. In addition, the average time for hemostatics usage in experiment group was 3.89 ± 0.33 days which was similar to that in control group (3.16 ± 0.20 days). The LOS for the experiment group was 5.92 ± 0.43 days and for control group was 6.30 ± 0.26 days. No significant difference was detected in the LOS between the 2 groups. Hospitalization expenses appeared to be similar between 2 groups. Postoperative bleeding rate and fever rate did not reach significant difference among control and experiment patients.

**Table 5:**
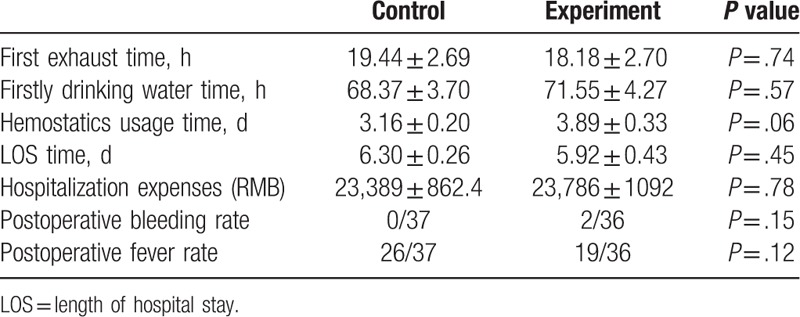
Clinical outcomes and postoperative complication after ESD surgery.

## 4 Discussion

Our study demonstrates that perioperative well-being of patients undergoing ESD is improved by the administration of POC. Our study has found that, as a result of oral carbohydrate in the preoperative period, patients have improved 3 out of 6 well-being parameters (thirst, hunger, and mouth dryness). This is in accordance with previous reports that found in open-heart surgery interventions, patients had effectively reduced thirst, as a main component in preoperative discomfort, when taking carbohydrate before surgery.^[20]^ Furthermore, it has also been observed that patients who received POC experienced a significant reduction in perioperative anxiety and hunger, as well as positive effects on muscular strength.^[21]^ However, in contrast to our data, as reported elsewhere, research reveals that there was no difference in the feeling of thirst for patients undergoing elective bowel resection.^[22]^ And, another study also shows in laparoscopic cholecystectomy no difference was found in postoperative sleep or well-being.^[23]^

Moreover, data from our study shows that VAS scores for nausea, vomit, and fatigue are not affected, neither before nor after surgery. Our data is contradictory to previous findings that indicate patients with carbohydrate intake have less nausea and vomiting compared with fasting patients.^[24]^ Hausel et al^[25]^ also found that in 172 patients undergoing laparoscopic cholecystectomy, POC may have a beneficial effect on the postoperative nausea and vomiting (PONV) 12 to 24 hours after surgery. We do not demonstrate this favorable effect of POC treatment on PONV in our study. This may be explained by fewer subjects included in our study. While, in line with our results, some studies also do not show any favorable effect of POC treatment on PONV in patients undergoing thyroidectomy and abdominal surgeries.^[7,26]^

Interestingly, we have found that patients with POC treatment have higher gastric sonography score which may indicate more gastric volume, while residual fluid collected by gastroscopy is similar between 2 groups. As we know, ultrasonography has several potential advantages including relatively convenience to perform, portability, noninvasiveness, and differentiating between fluid and solid gastric contents.^[27]^ We used a 3-point grading system (grades 0, 1, and 2) based on qualitative sonographic assessment of the gastric antrum that may correlate well with predicated gastric volume. However, this new proposed diagnostic tool has not yet been fully developed or validated and its diagnostic accuracy, reproducibility, and strength remain to be determined.^[28]^ A quantitatively mathematical model needs to be further validated by an independent method to measure gastric volume. Therefore, before being widely applied to clinical practice, gastric sonography grade as a predictor of aspiration needs to be further validated and characterized. Similar to our study, other studies report no occurrence of drink-related pulmonary complications in patients receiving POC treatment,^[24]^ suggesting the safety role of POC applied in ERAS protocol without increasing aspiration risk.

On the other hand, our results indicate that avoiding preoperative fasting with POC can decrease the degree of gastric peristalsis that may facilitate the successful completion of ESD surgery. The previous study has focused on the effects of POC treatment on surgical stress response, postoperative endocrine response, nutrition, and muscle function. Our study provides a new clue about the effect of POC on stomach peristalsis, further strengthening the beneficial outcome of POC treatment for patients undergoing ESD surgery. As we know, the continuous mucus layer covering the gastric mucosa functions as a barrier between luminal contents and the mucosa and exhibits gastroprotective roles. Meanwhile, the mucus barrier is important in protecting the gastric mucosa from mechanical friction and surgical injury, contributing to decreased gastric peristalsis.^[29,30]^ However, under stress state and fasting condition, the secretion of gastric mucus is decreased that could result in increased gastric peristalsis.^[31]^ Hence, it is plausible to speculate the finding that POC reduces gastric peristalsis in our study may be due to increased secretion of gastric mucus compared to patients with in-routine preoperative fasting. Further study is needed to investigate the detailed mechanism of the decreased gastric peristalsis induced by POC.

In addition, our research shows that POC treatment has no impact on length of hospital study and expense and other clinical outcomes including first time exhaust, first time for drinking water, and the usage of hemostasis. Similar to our results, Mathur et al^[9]^ reported no difference in the degree of fatigue or length of hospital study after major abdominal surgery. In contrast to our results, others found that patients undergoing cholecystectomy had shorter hospital stay by the intake POC.^[21]^ In contrast this study is limited by small sample size and small number of patients per group. Large multicenter RCTs are needed to further strengthen the evidence on the influence of POC on outcomes after ESD surgery.

## 5 Conclusions

Our study suggests that for patients undergoing ESD operation, perioperative feelings of thirst, hunger, and mouth dryness show significant improvement by preoperative oral intake of carbohydrates. Avoiding preoperative fasting with POC can decrease the degree of gastric peristalsis that may facilitate the successful completion of ESD surgery. Other aspects studied showed no significant differences regardless of patient convalescence, the length of hospital stay, and expenses.

## Author contributions

**Conceptualization:** Feng Ji.

**Data curation:** Zhenqiang Zhu.

**Investigation:** Hui Li.

**Methodology:** Yaqi Sun, Baoli Cheng.

**Supervision:** Xiangming Fang.

**Writing – Original Draft:** Yan Wang.

**Writing – Review & Editing:** Guohao Xie.
